# Bio-imaging: late gadolinium enhancement in hypertrophic cardiomyopathy and its relation to novel biomarkers of fibrosis

**DOI:** 10.1186/1532-429X-14-S1-P164

**Published:** 2012-02-01

**Authors:** Hassan Abdel-Aty, Hugo A Katus, Henning Steen, Stephanie Lehrke

**Affiliations:** 1 Department of Cardiology, University of Heidelberg, Heidelberg, Germany; 2 Charité, Berlin, Germany

## Background

Late gadolinium enhancement (LGE) accurately detects myocardial fibrosis in hypertrophic cardiomyopathy (HCM), which has been recently shown to be an independent risk factor for cardiac events in this setting. Endoglin is an accessory protein for the TGF-β receptor, which stimulates myocardial fibrosis through modulating the response to Angiotensin II. We sought to explore the relationship between the myocardial fibrosis (tissue substrate) as identified by LGE and Endoglin (biomarker) in the setting of HCM.

## Methods

We studied 133 HCM patients (56±16 y, 94 men) using a whole body 1.5 T CMR scanner (Philips Achieva) with 32 channel image aquisition. Vector-ECG gated short axis, two and four chamber cine slices with parallel image aquisition covering the entire left ventricle (LV) were acquired using a regular SSFP sequence. Matched LGE images were acquired after IV Gadolinium DTPA (0.2mmol/kg bodyweight) in all image planes. Two independent observers assessed LV volumes and myocardial mass that were acquired by manual drawing of endo- and epicardial borders. LGE was defined as signal intensity increase of >2SD of normal myocardium and after semi-automatic delineation on a commercially available workstation (Philips Viewforum) areas were quantified and expressed as percentage of the previously measured LV mass. Obstruction was determined using echocardiography gradient measurements at rest and under Valsalva.

P<0.05 was considered statistically significant.

## Results

79 patients (59%) exhibited intramural areas of LGE which averaged 7±12% of the LV mass. Patients with LGE had significantly higher levels of Endoglin compared with patients with no LGE lesions (3.8±0.4 vs. 2.4±0.3 nl, p=0.018). There was a weak but almost significant correlation between percent of LGE and Endoglin (r=0.16, p=0.06). Endoglin levels however were not related to obstruction (HNCM: 3.7±0.4 vs. 2.8±0.5, p=ns) and were not related to age, gender or LV mass.

## Conclusions

Our results provide preliminary evidence that Endoglin, a pro-fibrotic glycoprotein may be involved in the pathogenesis of late enhancement i.e. myocardial fibrosis in HCM. This molecular mechanism can have relevant clinical implications on the therapy of these patients since Endoglin expression (and thus probably myocardial fibrosis) can be suppressed using Angiotensin inhibitors although further studies on the prognosis and risk stratification of this new bio-imaging parameter have to be conducted.

## Funding

None.

